# A Molecularly Imprinted Polypyrrole/GO@Fe_3_O_4_ Nanocomposite Modified Impedimetric Sensor for the Routine Monitoring of Lysozyme

**DOI:** 10.3390/bios12090727

**Published:** 2022-09-05

**Authors:** Pablo Montoro-Leal, Isaac A. M. Frías, Elisa Vereda Alonso, Abdelhamid Errachid, Nicole Jaffrezic-Renault

**Affiliations:** 1 Department of Analytical Chemistry, Faculty of Sciences, University of Málaga, 29016 Málaga, Spain; 2 Institut des Sciences Analytiques, University of Lyon, 69100 Villeurbanne, France

**Keywords:** molecularly imprinted polymer, decorated graphene oxide, lysozyme, electrochemical impedance spectroscopy

## Abstract

Lysozyme (LYS) applications encompass anti-bacterial activity, analgesic, and anti-inflammatory effects. In this work, a porous framework that was based on the polymerization of pyrrole (PPy) in the presence of multi-functional graphene oxide/iron oxide composite (GO@Fe_3_O_4_) has been developed. Oxygen-containing and amine groups that were present in the nanocomposite were availed to assembly LYS as the molecularly imprinted polymer (MIP) template. The synthesized material (MIPPy/GO@Fe_3_O_4_) was electrodeposited on top of a gold microelectrode array. Transmission electron microscopy (TEM) and X-ray photoelectron spectroscopy (XPS) were used to confirm the adequate preparation of GO@Fe_3_O_4_, and the characterization of the resulting molecularly imprinted electrochemical sensor (MIECS) was carried out by electrochemical impedance spectrometry (EIS), FT-IR analysis, and scanning electron microscopy (SEM). The impedimetric responses were analyzed mathematically by fitting to a Q(Q(RW)) equivalent circuit and quantitative determination of LYS was obtained in a linear range from 1 pg/mL to 0.1 µg/mL, presenting good precision (RSD ≈ 10%, n = 5) and low limit of detection (LOD = 0.009 pg/mL). The fabrication of this device is relatively simple, scalable, rapid, and economical, and the sensor can be used up to nine times without disintegration. The MIECS was successfully applied to the determination of LYS in fresh chicken egg white sample and in a commercial drug, resulting in a straightforward platform for the routine monitoring of LYS.

## 1. Introduction

Lysozyme (LYS) is a globular protein of relatively low molecular weight (14 kDa) that can be found in biological fluids such as tears, saliva, and milk [1]. The structure of this protein consists of two regions, an external hydrophilic surface and an internal hydrophobic core. LYS has been considered as a potential biomarker for the diagnosis of leukemia and other diseases that cause damage in human cells, tissues, and organs [2]. Moreover, due to its anti-bacterial activity, analgesic, and anti-inflammatory effects, thermotolerance, and chemical stability, LYS is used in pharmacology as a functional ingredient for medical treatments and in the food industry as a preservative [3,4].

LYS determination has been performed through high-performance liquid chromatography (HPLC), mass spectrometry (MS), enzyme-linked immunosorbent assay (ELISA), fluorescence, and electrophoresis [5,6,7,8,9]. However, in most cases, long processing times, complex sample pre-treatments, and expensive equipment are required. As an alternative, biosensors are “ready to use” devices that are designed to be simple analytical methods with increased sensitivity, rapid response, and real-time analysis [2,10,11]. It should be noted that electrochemical biosensors feature electronic components with miniaturization and automation that are defined by the electronic sensitivity of the transducer technique.

Molecular imprinting technology is a technique for the preparation of polymer-based sensors. In this type of synthesis, the functional monomer is polymerized in the presence of the target molecule (template). After polymerization, the polymeric functional groups are correctly oriented to allow interactions with the template molecule which remains adsorbed to the material. Finally, the template is extracted using the necessary electrochemical or chemical conditions to break the existing interactions between the template and the polymer. As a result, specific three-dimensional sites with a similar size and shape to the template are created [12] and used as specific detection sites for the analyte. Molecularly imprinted polymers (MIPs) are usually applied for the development of selective analytical methods [13,14]. The detection of viruses, proteins, cells, nucleic acids, drugs, and inorganic ions has been successfully performed [15,16,17]. The use of molecular imprinting technology has been extended to numerous research fields because of its advantages. Frequently, high porous surface structures are wanted to increase the surface to volume ratio and thus, achieve increased recognition [18]. The nature and number of interactions between the monomer and the molecular template during the polymerization process are essential factors in the performance of MIPs because the formation of stable complexes results in more efficient imprinted sites. The capacity to establish hydrogen bonds and dipole interactions through functional groups containing oxygen and nitrogen is highly desired for protein detection applications [19]. On the other hand, employing MIPs as transducers requires the transduction of the capturing event into a quantifiable electronic output signal. In this regard, polypyrrole (PPy) presents high electron-transfer capacity, stability, and low toxicity. Ppy interacts with organic and inorganic molecules by hydrophobic interactions via its extended aromatic structure and dipole moment [20]. However, the electrons of the imine group are not available to establish efficient hydrogen bonds. Consequently, many authors usually propose the use of Ppy in combination with alternative polymers and additives such as o-phenylenediamine [21], chitosan [22], and carboxylic acids [23] to increase the number and diversity of interactions between the imprinted polymer and the template.

Some well-known nanomaterials such as gold nanoparticles, iron oxide nanoparticles (Fe_3_O_4_-NPs), graphene, and graphene oxide (GO) have been used to improve the sensitivity and selectivity of electrochemical sensors [24,25,26]. Of note, GO is an interesting 2D aromatic structure material with high specific surface area (2.630 m^2^/g), and numerous oxygen-containing functional groups on the basal plane and the sheet edge, including epoxy, carboxylic acid, carbonyl, and hydroxyl groups [27]. Despite the presence of the π-π domain, a hydrophilic character is provided, resulting in high adsorption capacity for the retention and preconcentration of polar species and metal ions. In recent years, researchers have proposed the coupling of Fe_3_O_4_-NPs and GO, combining their biocompatibility, degradability, physiological and chemical stability, and excellent adsorbent properties for the preparation of decorated graphene oxide (GO@Fe_3_O_4_) [28,29]. For this reason, in this work, GO@Fe_3_O_4_ nanocomposite was selected, presenting amine groups and oxygen-containing functional groups that are compatible to interact with the template through dipole interactions and hydrogen bonds, and used to produce microporous polypyrrole (MIPPy/GO@Fe_3_O_4_) imprinted with lysozyme. Pyrrole is interesting for scaling up production because of its low cost and easy polymerization reaction [30,31,32]. The inclusion of a multi-functional composite for the routine monitoring of lysozyme resulted in extraordinary sensitivity and selectivity. In this MIECS, the complex interactions with the template are originated from the hydrophobic interactions and dipole moment of PPy as well as from the oxygen- and nitrogen-containing functional groups of GO@Fe_3_O_4_ which are crucial to create the specific sites. The MIECS was successfully applied for the monitoring of LYS in chicken egg and commercial drugs. Thus, in this work, the synthesized MIECS is presented as an easy to use and reliable alternative methodology for the determination of lysozyme in the pharmaceutical industry.

## 2. Materials and Methods

### 2.1. Reagents and Samples

All the aqueous solutions were prepared with analytical grade reagents and doubly deionized water (DDW) that was purified with an ELGA Purelab Classic Ultrapure Water System (High Wycombe, Bucks, UK) that reaches a resistivity of 18.2 MΩ·cm at 25 °C. Ferrous chloride tetrahydrate (FeCl_2_·4H_2_O), ferric chloride hexahydrate (FeCl_3_·6H_2_O), hydrochloric acid 37%, ammonium hydroxide 30%, hydrogen peroxide 30%, graphite powder, methanol, ethanol, sodium chloride, potassium permanganate, tetraethyl orthosilicate ≥98%, 3-aminopropyltriethoxysilane (AP) ≥ 99%, and sulfuric acid ≥ 99% from Merck (Darmstadt, Germany) were used. N,N’-dicyclohexylcarbodiimide (DCC), pyrrole ≥ 98%, hexadecyl trimethyl ammonium bromide (HTMA) ≥ 99%, sodium dodecyl sulphate (SDS) ≥ 98%, phosphate-buffered saline (PBS), lysozyme from chicken egg white, bovine serum albumine (BSA), and peroxidase were purchased from Sigma Aldrich (St. Louis, MO, USA).

### 2.2. Instrumentation

Electrochemical experiments were performed using a multichannel potentiostat analyzer PalmSens4 multiplexer MUX8 acquired from PalmSens BV (Houten, The Netherlands). The microelectrodes, fabricated at the National Center for Microelectronics (CNM, CSIC, Sevilla, Spain), were designed over a silicon chip to produce an array of four gold working electrodes (WE, 0.64 mm^2^/unit), two Ag/AgCl reference microelectrodes (RE, 1.37 mm^2^/unit) and one counter microelectrode (CE, 0.13 mm^2^). Prior to use, the surfaces of the microelectrodes were cleaned in an air-plasma treatment at 0.2 mBar and 30 s of application.

X-ray photoelectron spectroscopy (XPS) analysis were performed with a Physical Electronics ESCA 5701 instrument (Chanhassen, Minnesota, USA); the binding energies (BE) were observed considering the position of the C1s peak at 284.8 eV. The residual pressure in the analysis chamber was maintained below 3 × 10^−9^ torr during data acquisition.

The microstructures of GO and GO@Fe_3_O_4_ were characterized by transmission electron microscopy (TEM) JEOL, JEM-1400 (Peabody, MA, USA). The surface morphology of the modified electrodes were observed by scanning electron microscopy (SEM) VEGA TESCAN SEM from Leica Microsystems (Wetzlar, Germany) at 15 kV. SEM analyses were carried out with gold metallization.

FT-IR NEXUS from ThermoFisher Scientific (Waltham, MA, USA) with the accessory Microscope Continuµm was used for the study of the surface composition of the modified microelectrodes in the 500–3200 cm^−1^ range and 256 scans.

### 2.3. Synthesis of GO@Fe_3_O_4_

FeCl_3_·6H_2_O (11.68 g) and FeCl_2_·4H_2_O (4.30 g) were mixed in 200 mL of DDW at 80 °C, and then 50 mL of 30% ammonia solution was added quickly in N_2_ atmosphere. In this moment, Fe_3_O_4_ nanoparticles were coprecipitated and the resulting black suspension was stirred for 75 min with reflux. The Fe_3_O_4_ nanoparticles were cooled to room temperature and washed with DDW. The synthesis of MNPs was optimized by González Moreno et al. [33], and the resulting Fe_3_O_4_ nanoparticles were coated with the following procedure. First, Fe_3_O_4_ nanoparticles were mixed with 8 mL of TEOS and 60 mL of glycerol in 200 mL of ethanol at 60 °C and stirred for 2 h in N_2_ atmosphere. The resultant suspension was cooled at room temperature and washed with DDW and ethanol. Then, the product was suspended in 150 mL of AP 1% prepared in ethanol 95%, and the pH was adjusted to 4.5. The mixture was stirred at 60 °C for 2 h in N_2_ atmosphere. Later, the suspension was cooled at room temperature and washed with DDW and methanol. All the washing steps were carried out with the aid of an external permanent magnet to separate the nanoparticles from the matrix thanks to the magnetic properties of Fe_3_O_4_. On the other hand, GO was prepared from natural graphite following the process that was described by Diagboya et al., being purified by centrifugation cycles [34]. Finally, 500 mg of GO was suspended in 50 mL of ethanol, containing 500 mg of coated Fe_3_O_4_ and 0.25 g of DCC in a 100 mL round-bottom flask. The mixture was sonicated for 10 min and kept at reflux at 50 °C for 48 h.

### 2.4. Synthesis of MIPPy/GO@Fe_3_O_4_

**Figure 1:**
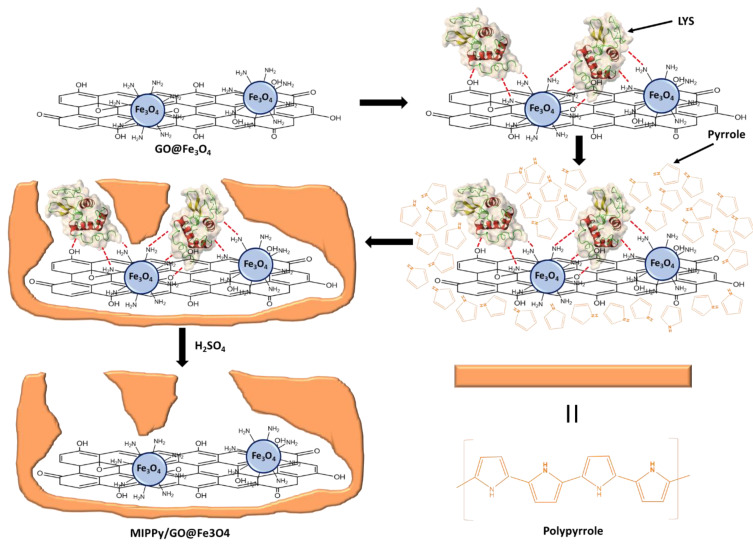
Synthesis process of MIPPy/GO@Fe_3_O_4_.

First, the polymerization solution was prepared as follows: 20 mg of SDS were dissolved in 8.3 mL of DDW. Then, 1 mg of GO@Fe_3_O_4_ was added to the prepared solution and the mixture was sonicated for 20 min. Next, 1 mL of the LYS 1 mg/mL solution was added to the resulting suspension, maintaining stirring for 20 min. Finally, 0.7 mL of pyrrole was added to the mix, maintaining stirring for 20 min. The addition of SDS in the polymerization solution presented a double function. (1) The presence of a surfactant contributed to the stabilization of the GO@Fe_3_O_4_ suspension, and (2) pyrrole is insoluble in aqueous solutions, so the surfactant was necessary for the preparation of pyrrole micelles. The final SDS concentration in the prepared solution was 2 mg/mL. Then, the microelectrodes were introduced in the polymerization solution, and the electrodeposition of polypyrrole was carried out by cyclic voltammetry (CV) between −0.6 V and 1.2 V, 1 scan with a scan rate of 100 mV/s at 5 °C. After electrodeposition, the microelectrodes were rinsed with DDW and dried under a weak nitrogen flow. In this work, SDS has been used during the synthesis process of MIPPy/GO@Fe_3_O_4_, which is a negatively charged surfactant. As a result, the surface of the prepared material could present residual negative charges due to the presence of SDS that was embedded in the polymerized structure. These negative charges and the dipole moment of PPy that are located on the surface could contribute to establish undesirable interactions with other proteins and molecules. For these reasons, a passivation step was necessary, for which a positively charged surfactant was selected. Thus, the modified surface was passivated by introducing the microelectrodes in HTMA 1 mg/mL, prepared in PBS, for 1 h. Finally, the last step for MIECS preparation consisted of the removal of the LYS template by incubating the microelectrodes in a 0.5 M H_2_SO_4_ solution for 1 h. The synthesis process can be observed in Figure 1 (the passivation step was omitted in order to simplify the scheme). There were two modifications that were carried out as a reference: the corresponding non-imprinted (NIPPy/GO@Fe_3_O_4_) modified microsensors were prepared analogously except for the absence of LYS template, and the imprinted polypyrrole (MIPPy) microsensors were prepared following an identical protocol without GO@Fe_3_O_4_.

### 2.5. Electrochemical Measurements

EIS (Higher Freq = 100 kHz, Lower Freq = 1 Hz, Eac = 0.05 V, Edc = 0.05 V) was used to characterize the microelectrode modification and perform the analysis of real samples. The MIP-modified microelectrodes were introduced in PBS solution containing LYS (from 0.01 to 1 105 pg/mL) for 30 min to study their analytical performance and the equivalent circuit of the system. A washing step with water was performed before measurements to eliminate the excess non-attached LYS on the surface. The influence of possible interferences in the signal (BSA and peroxidase) was studied under the same experimental conditions and concentration range. Finally, the value of the impedance at 1050 Hz was selected as the analytical signal for the determination of LYS in fresh chicken egg white and in a commercial drug.

## 3. Results and Discussion

### 3.1. Morphological Characterization and Composition

TEM images were used to study the morphology of GO and GO@Fe_3_O_4_. In Figure S1A (Supplementary Material), the 2D structure of GO can be clearly observed, according to the information that was found in bibliography. Besides, Figure S1B shows the presence of Fe_3_O_4_ nanoparticles scattered over the GO layer, indicating that both materials were adequately coupled. The XPS spectrum of GO@Fe_3_O_4_ is shown in Figure S2, where the peaks of Fe, O, N, C, and Si were assigned. The presence of N, Fe, and Si in the material resulted from the silica-coated Fe_3_O_4_ nanoparticles, while the presence of C was related to the composition of GO. The high O content was justified by the composition of both GO and Fe_3_O_4_ nanoparticles. Therefore, TEM and XPS analysis indicated that GO@Fe_3_O_4_ was adequately prepared.

**Figure 2:**
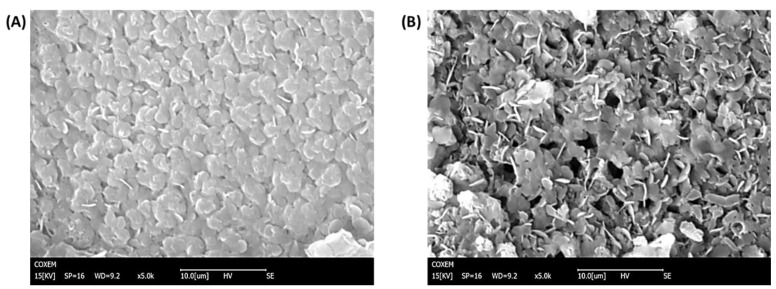
SEM images of (**A**) NIPPy/GO@Fe_3_O_4_ (**B**) MIPPy/GO@Fe_3_O_4_.

**Figure 3:**
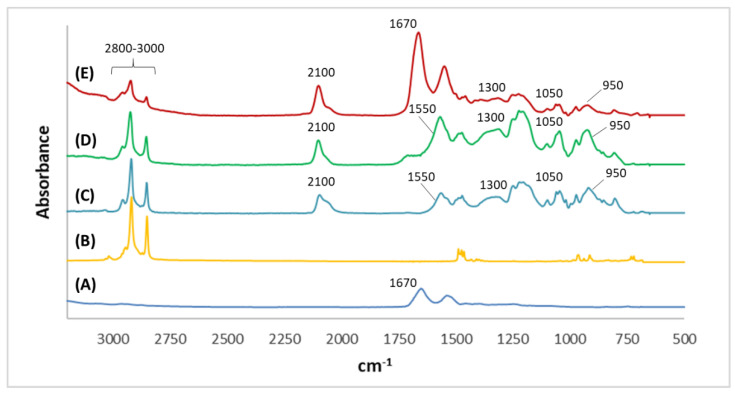
Infrared spectra in the 3200–500 cm^−1^ range of (**A**) LYS, (**B**) HTMA, (**C**) NIPPy/GO@Fe_3_O_4_, (**D**) MIPPy/GO@Fe_3_O_4_, and (**E**) MIPPy/GO@Fe_3_O_4_ incubated in LYS.

SEM was performed to investigate the morphology of the microelectrodes modifications. As can be seen in Figure 2A, the non-imprinted film presented a more compact structure in comparison with the imprinted film, which presented more roughness and wrinkles (Figure 2B). Moreover, nanoholes can be observed in the structure of MIPPy/GO@Fe_3_O_4_. FT-IR analysis was carried out to confirm the passivation step in the preparation of MIPs, and the LYS retention on the surface of the modified microelectrodes after 30 min of incubation. In the LYS spectrum (Figure 3A), a strong peak at 1670 cm^−1^ can be attributed to the stretching vibration C = O of amino acids. From FT-IR spectra of HTMA (Figure 3B), several characteristic bands can be seen in the 2800–3000 cm^−1^ range that were assigned to C-H stretching, usually found in alkylammonium cations [35]. As can be observed in Figure 3C,D, NIPPy/GO@Fe_3_O_4_ and MIPPy/GO@Fe_3_O_4_ presented the same infrared profile (number of bands, position, and intensity), indicating that the chemical composition of both materials is very similar. The bands at 950 cm^−1^ were assigned to aromatic C-H from GO, and the signals at 1050 cm^−1^ and 2100 cm^−1^ were assigned to νC-N and νSi-H [36], according to the presence of aliphatic amines and silicon species from the coated Fe_3_O_4_ nanoparticles. In NIPPy/GO@Fe_3_O_4_ and MIPPy/GO@Fe_3_O_4_ spectra, characteristic bands of HTMA appeared in the 2800–3000 cm^−1^ range, so the passivation step was adequately performed when imprinted and non-imprinted materials were prepared. Moreover, characteristic bands of PPy were observed at 1550 cm^−1^ (pyrrole ring), and 1300 cm^−1^ (plane = C–H) [37]. Besides, when the MIECS was incubated in LYS 1 105 pg/mL solution for 30 min, an additional band appeared at 1670 cm^−1^ corresponding to the presence of LYS on the surface (Figure 3E).

### 3.2. Fabrication Optimization

During pyrrole electrodeposition, the scan rate (V/s) and the final concentration of SDS (mg/mL) were considered as important synthesis parameters to be optimized due to their influence on the morphology and stability of the modified surface. The selected parameters were optimized by using a univariant strategy, changing one parameter and keeping the other constant. For scan rate optimization purposes, the SEM images were observed to ensure the most homogeneous modified electrode surface as possible. The synthesis of the modified microelectrodes was performed in the 0.01–0.15 V/s scan rate range. As can be observed in Figure S3, the modified surfaces with non-homogeneous structures were obtained when the scan rate was lower than 0.15 V/s (Figure S3A), while a more homogeneous surface was obtained with the maximum scan rate value (Figure S3B). So, 0.15 V/s was selected as the optimum scan rate. The SDS concentration that was used during MIPPy/GO@Fe_3_O_4_ synthesis was varied between 0.5 and 5 mg/mL, and it was observed that the MIPPy film was easily detached from gold microelectrode surfaces during the washing steps when the SDS concentration was higher than 2 mg/mL. Therefore, 2 mg/mL was selected as the optimum SDS concentration in order to avoid instability problems of the MIPPy film. In this work, the acronym MIECS is used to refer to the modified microelectrodes with MIPPy/GO@Fe_3_O_4_ under optimum conditions.

### 3.3. Impedimetric Characterization

For the impedimetric measurements, a small AC/DC signal was applied to reduce the signal noise and both parameters were studied in the 0–0.1 V potential range. To perform these experiments, gold microelectrodes were modified with MIPPy/GO@Fe_3_O_4_, introducing the prepared MIECS in PBS solution. The signal noise of EIS graphs was reduced when the AC/DC values increased from 0 to 0.1 V. However, when 0.1 V was used, the integrity of the sensors was negatively affected due to the excess voltage. Therefore, medium values were finally selected (Eac = 0.05 V, Edc = 0.05 V) to avoid serious damage in the electronic system.

**Figure 4:**
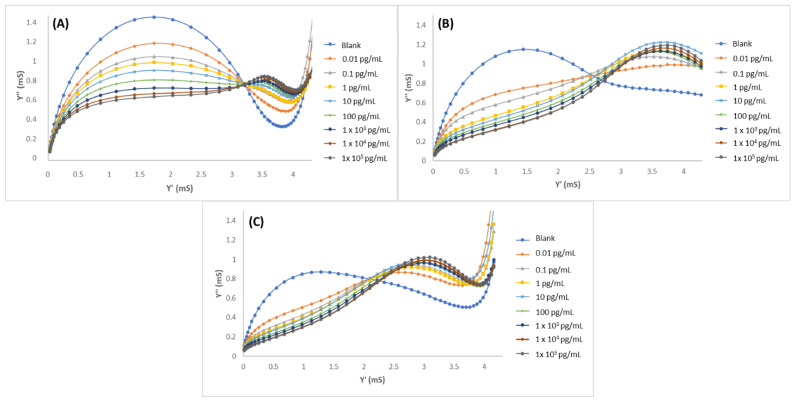
Admittance spectra of (**A**) MIPPy/GO@Fe_3_O, (**B**) NIPPy/GO@Fe_3_O_4_, and (**C**) MIPPy, after incubation with LYS in 0.01–1 × 10^5^ pg/mL range.

Gold microelectrodes were modified with MIPPy/GO@Fe_3_O_4_, and the LYS concentration was determined by performing EIS measurements in PBS 10 mM, pH 7.4. Standard LYS solutions from 1 × 10^−2^ to 1 × 10^5^ pg/mL were measured. The resultant admittance spectra were plotted as the susceptance (imaginary part of admittance) vs. the conductance (real part of the admittance) and are shown in Figure 4. In the graphics (Figure 4A), a clear tendency of decreasing susceptance can be observed in relation to the increment of LYS concentrations. In these graphics, a first semicircle can be observed (from left to right) corresponding to a relaxation at lower frequencies and a second relaxation (on the right) corresponding to high frequencies. Molecular movements at lower frequencies are characterized by the ionic diffusion time of the redox probe, while the response that was obtained at higher frequencies is associated with the accumulation of target molecules over the biosensor surface. MIPPy/GO@Fe_3_O_4_ (Figure 4A) demonstrated a good correlation between the LYS concentration and the variation of the admittance signal in the studied concentration range. For comparison, experiments were performed under the same conditions but using two alternative modifications: the same preparation process but eliminating the GO@Fe_3_O_4_ from the synthesis process (MIPPy) and maintaining the synthesis process without the template (NIPPy/GO@Fe_3_O_4_). The EIS spectra corresponding to the experiments that were performed with NIPPy/GO@Fe_3_O_4_ and MIPPy (without modifiers) are observed in Figure 4B,C, respectively. At first, susceptance decreased with the increment of LYS concentrations, but rapidly saturated when the LYS concentration exceeded 10 pg/mL. This fact indicated that the presence of GO@Fe_3_O_4_ during the synthesis process greatly improved the performance of the sensor. Moreover, it can be concluded that the response of the NIPPy/GO@Fe_3_O_4_ microsensor toward LYS is very weak in comparison with the response of the MIPPy/GO@Fe_3_O_4_, demonstrating that MIP was successfully prepared.

**Figure 5:**
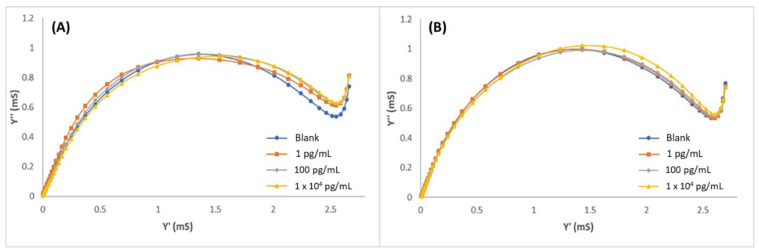
Admittance spectra of MIPPy/GO@Fe_3_O after incubation with (**A**) peroxidase and (**B**) BSA in 1–1 × 10^4^ pg/mL range.

Selectivity was evaluated towards the potential protein interferences by using MIPPy/GO@Fe_3_O_4_ as previously described for MIECS fabrication. All the measurements were performed by the EIS technique after 30 min incubation under optimized experimental conditions. Peroxidase and BSA were selected as interferences, and solutions of both proteins were prepared in PBS with three different concentrations: 100 fg/mL, 10 pg/mL, and 10 ng/mL. The admittance spectra of peroxidase and BSA solutions are shown in Figure 5A,B, respectively. No tendency was observed in the graphics, and no correlation could be stablished between the interference concentration and the signal. This indicated that BSA or peroxidase were not adsorbed on the MIECS surface, demonstrating that the system is very selective towards the target molecule.

### 3.4. Equivalent Circuit Model Analysis and Analytical Performance

**Figure 6:**
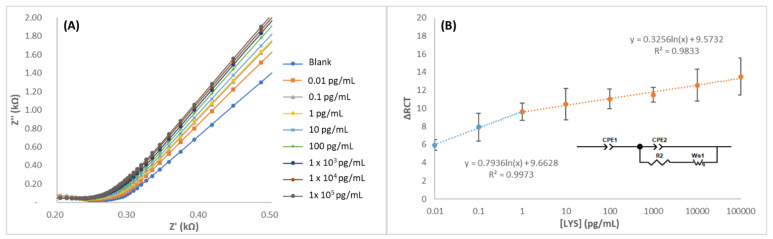
(**A**) Nyquist spectra of MIPPy/GO@Fe_3_O after incubation with LYS in 1–1 × 10^5^ pg/mL range and (**B**) the equivalent circuit and calibration curve that was obtained from the fitting process.

The LYS solutions were prepared in PBS in the 0.01 to 10^5^ pg/mL range and analyzed by EIS. Impedance spectroscopy was used to evaluate the charging behavior of the electrical double layer and interfacial relationships between lysozyme and the transducer. Nyquist impedance plots were recorded to inspect the fabrication process and performance of the MIEC. The impedimetric responses were mathematically fitted to an equivalent circuit consisting of the solution resistance (RS) in series with a constant phase element (CPE) in parallel with a charge transfer resistance (RCT) and a Warburg impedance (W). The interactions occurring at the electrode surface hinder or favor electronic transfer and/or increase the impedance. For this reason, CPE and RCT depend on the dielectric and isolating aspects of the interface of the biosensor. These analytical signals and respective RCT values were directly proportional to the concentration of the targeted lysozyme. In supplementary Table S1, the average results that were obtained through the equivalent circuit for three different electrodes are shown, including MIECS with LYS, BSA, and peroxidase. A good correlation when MIECS was subjected to LYS-positive control samples was observed, where the ΔRC values increased gradually in a linear relation to the concentration of LYS present in the sample (Figure 6). However, in the case of negative controls samples (without LYS), it can be observed that no correlation is achieved attending to the concentration increase. This effect can be explained considering the small size of LYS in comparison to the other proteins. It should be noted that the extraction of the template promotes a heterogeneous porous surface as can be observed in the morphological characterization. This new morphology in combination to the size of the other proteins (around 40K) promotes the unspecific binding and layer sorption and desorption that was observed by impedance spectroscopy (Figure 6). There were two different linear ranges that were observed before and after the concentration of 1 pg/mL. The calibration curve was defined as y = a + b·log(x), where the parameter “b” was the slope of the calibration curve, “a” was the intercept, and Sa was the intercept uncertainty (considered as the standard deviation of the blank). The first region, from 10 fg to 1 pg was adjusted to the following equation ΔRCT = 9.635 + 0.797 log(C_LYS_) with r^2^ = 0.99, with an instrumental limit of detection, LOD = 2 fg (where Sa = 0.07). The second linear region was fitted to the following equation ΔRCT = 9.509 + 0.315 log(C_LYS_) with r^2^ = 0.91. From this study was also concluded that the sensor can be used up to nine times without disintegration.

**Figure 7:**
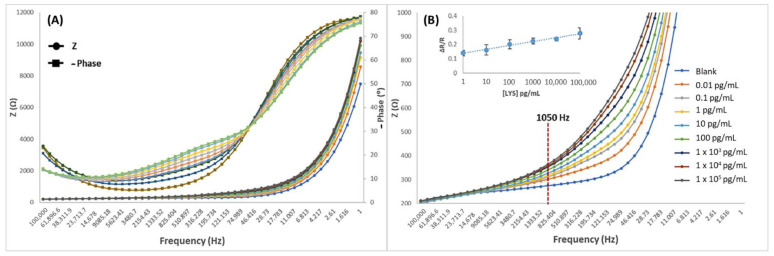
Bode spectra of MIPPy/GO@Fe_3_O after incubation with LYS in the 1–1 × 10^5^ pg/mL range. X axis: frequency 1–100,000 Hz and Y axis: Z (**A**) 0–12,000 Ω and (**B**) 200–1000 Ω. (**B**) includes the resulting calibration curve that was obtained after representation of the 1050 Hz data.

In order to develop a routine analytical method for LYS monitoring according to the usual working range of portable potentiostats that are available on the market (>1000 Hz), the Bode plots were analyzed (Figure 7A). As can be observed in Figure 7A, the rates of phase angle shifts between 100,000 and 46 Hz have greater variation rate in the range from 0 to 100 pg/mL, than in the 10^3^ to 10^5^ pg/mL concentration range, where the rate rapidly decreases; this phenomenon can be better understood from the admittance graphics (Figure 4). At these frequencies, the diffusion process of the redox probe is largely hindered by the captured biomolecules and, therefore, after concentrations of 100 pg/mL, the diffusion of the redox probe becomes irrelevant, and the response appears to be driven by the capacitive accumulation. In Figure 7B, the developed MIP transducer presented a good distinguishable response at frequencies from 1 to 1050 Hz; where 1050 Hz was finally selected, and its corresponding impedance values were used as an analytical signal. Supported in Rct values, the Bode data were normalized by calculating ΔR/R as ΔR/R = (Rct LYS − Rct blank)/Rct blank), presenting the following linear calibration: y = 0.0117 × Ln (C_LYS_) + 0.1408 with r^2^ = 0.983. The blank signal corresponds to the negative control without LYS. LOD and LOQ were defined as the concentration value corresponding with a signal 3.3 and 10 times the uncertainty of the blank, respectively. The calibration curve was defined as y = a + b·ln(x), where the parameter “b” was the slope of the calibration curve, “a” was the intercept, and Sa was the intercept uncertainty (considered as the standard deviation of the blank). For the impedimetric microsensor that was based on MIPPy/GO@Fe_3_O_4_, LOD = 0.009 pg/mL and LOQ = 0.9 pg/mL (Sa = 0.008) were obtained.

Attending to the reusability of the system, an additional wash step with H_2_SO_4_ was performed after the LYS 10 pg/mL measurement in order to remove the excess protein from the surface of the MIECS. Then, the incubation and the measurement of LYS was performed and this cycle was repeated two more times. The measurements were obtained adequately but the original impedance value was not achieved, decreasing by 50% and remaining constant (Figure S4). Therefore, it can be concluded that the sensor can be reused, but the sensitivity of the method is compromised.

For the evaluation of the reproducibility of the method in a wide linear range, three gold microelectrodes were used to measure two standard LYS solutions (1 pg/mL and 1000 pg/mL), employing the remaining third as a NIP blank control. The relative standard deviations (RSDs) for both concentrations were calculated, providing a 7% (n = 5) and 11% (n = 5), respectively.

In Table 1, a comparative study of the analytical performance of the proposed microsensor and that of previous electrochemical methods that were reported in the bibliography for the determination of LYS can be observed [2,22,38,39,40]. To the best of our knowledge, the proposed MIEC demonstrated a more extended linear range (five orders of magnitude) and a very lower LOD than the reported sensors, presenting the best analytical performance. In a previous work, our group showed the feasibility of using a chitosan-based MIP to detect lysozyme. At the time, PPy was used as a supporting layer to attach the electrodeposited MIP chitosan film and the attained LOD was 70 pg/mL.

Molecular imprinted polymers (MIPs) require the polymerization of monomers with appropriate functional groups to favor the complexation of a given template. In the present work, the MIP was prepared directly over the PPy layer in the presence of a multifunctional composite which presents diverse functional groups to bind lysozyme. Other works have reported to improve lysozyme detection in differential pulse voltammetry sensors by including CuFe_2_O_4_ magnetic nanospheres in glassy carbon to improve its capacitance characteristics and conductivity. However, despite its good selectivity, its working detection range is of 50–800 ng/mL and a detection limit of 1.5 ng/mL. To the best of our knowledge, the enhancement that was found in the present PPy/GO@Fe_3_O_4_ MIP arises from the interactions between the imprinted composite and the template, the synergy occurring between PPy π-electron conjugated framework and semiconducting GO@Fe_3_O_4_.

### 3.5. Application

To validate the proposed microsensor for its potential application, MIECS was used for the determination of LYS in a fresh chicken egg white sample and commercial drug. The impedance value that was obtained under optimum conditions at 1050 Hz was used as an analytical signal, and the accuracy of the method was studied using spike tests in the real samples. A tablet of commercial drug was dissolved in 10 mL of DDW, and both the commercial drug solution and fresh chicken egg white sample were diluted ten million times prior to analysis. The samples were analyzed by standard addition calibration in the 10–100 pg/mL range. As can be observed in Table 2, the preparation and initial samples dilution were considered, and all the recoveries were close to 100% for all the spiked samples. Moreover, the LYS concentration that was found in the fresh chicken egg white sample and commercial drug were 4.8 ± 0.8 mg/mL and 23 ± 4 mg/tablet, according to the expected concentration of LYS in chicken egg white (about 3.5 mg/mL) [41] and the LYS concentration value that was indicated on the label of the commercial drug (20 mg/tablet), respectively.

## 4. Conclusions

A novel sensitive impedimetric microsensor has been developed for lysozyme determination by using a novel molecularly imprinted polymer that was based on PPy/decorated graphene oxide (MIPPy/GO@Fe_3_O_4_) composite. The adequate preparation of the material was verified by several analytical techniques: TEM and XPS were applied for the study of the composition and morphology of GO@Fe_3_O_4_, while SEM and FT-IR were used to characterize the modified microelectrode surface. EIS was used to investigate the electrochemical behavior of the system, obtaining an equivalent circuit to analyze each component of the modified microsensors. The electrochemical characterization of the sensors indicated that GO@Fe_3_O_4_ was adequately deposited on the microsensor surface, and the presence of this material during the synthesis process greatly improved the performance of the system. The MIECS showed an extended linear range with a very low detection limit, good reproducibility, and suitable precision. This sensor has proven to be not susceptible to interferences versus other proteins thanks to the specific recognition sites that were generated. Besides, several synthesis and measurement parameters were adequately optimized by following a univariant strategy. In this work, the analytical performance was studied under optimum conditions and compared with that of other electrochemical methods that were previously reported in the bibliography, presenting one of the best analytical performances for the routine monitoring of LYS. Although the LYS concentration ranges are usually high and out of the working range of the sensor, biological samples present complex matrices that can contribute to the non-suitable performance of an electrochemical biosensor due to matrix effects and the presence of interferents. In this work, the use of more diluted samples in order to avoid matrix effects is possible, improving the performance of the sensor even in complex matrices. The prepared MIECS have been successfully applied for the detection of LYS in fresh chicken egg white and commercial drugs, obtaining high recovery values.

## Figures and Tables

**Table 1:** Comparison of the analytical performance of the fabricated MIPPy/GO@Fe_3_O_4_-based microsensor with previously published sensors for LYS detection.

Functional Polymer	Method	Linearity (ng/mL)	LOD (ng/mL)	Ref.
NIPAM ^a^, AAM ^b^, MAA ^c^	Differential pulse voltammetry	50–800	1.54	[2]
NIPAM, AAM	Fluorescence	980–9800	490	[38]
AAM, MAA, DMAEMA ^d^	Fluorescence	0–2.5 × 10^4^	1400	[39]
-	SPR ^e^	10^4^–10^6^	0.5 × 10^4^	[40]
CS ^f^	EIS	0.3–5 × 10^4^	0.07	[22]
PPy/GO@Fe_3_O_4_	EIS	0.001–100	9 × 10^−6^	This work

^a ^N-isopropylacrylamide. ^b^ Acrylamide. ^c^ Methyl methacrylate. ^d^ Dimethylaminoethyl acrylate. ^e^ Surface plasmon resonance. ^f^ Chitosan.

**Table 2:** Analysis that was performed for the determination of LYS in real samples.

Sample 1	Added (mg/mL)	Found (mg/mL)	Recovery (%)
Chicken egg white	-	4.8 ± 0.8	-
	6	12.1 ± 0.7	112
	16	20.3 ± 0.8	98
**Sample 2**	**Added (mg/tablet)**	**Found (mg/tablet)**	**Recovery (%)**
Commercial drug	-	23 ± 4	-
	30	52 ± 14	97
	100	122 ± 5	99

## Supplementary Materials

The following supporting information can be downloaded at: https://www.mdpi.com/article/10.3390/bios12090727/s1, Figure S1: TEM images of (A) GO (scale of 200 nm), and GO@Fe_3_O_4_ with a scale of (B) 200 nm, (C) 100 nm, and (D) 50 nm; Figure S2: XPS spectra of GO@Fe_3_O_4_ and atomic composition; Figure S3: SEM images of PPy that was synthesized by electropolymerization (A) prior to scan-rate optimization and (B) under scan-rate optimized conditions (0.15 V/s); Figure S4: Reusability study results after three LYS 10 pg/mL incubation cycles; Table S1: Average data for equivalent circuit of MIPPy/GO@Fe_3_O_4_ impedimetric responses.
