# Supplementary material for: A Molecularly Imprinted Polypyrrole/GO@Fe3O4 Nanocomposite Modified Impedimetric Sensor for the Routine Monitoring of Lysozyme
Source: Biosensors (Basel). 2022 Sep 5;12(9):727. doi: 10.3390/bios12090727 (PMC9496344; doi:10.3390/bios12090727)
Supplement: Supplementary file 1 [file biosensors-12-00727-s001.zip › biosensors-1866640-supplementary.pdf]

A Molecularly Imprinted Polypyrrole/GO@Fe_3O_4 Nanocomposite Modified Impedimetric Sensor for the Routine Monitoring of Lysozyme

Pablo Montoro-Leal, Isaac A. M. Frías, Elisa Vereda Alonso, Abdelhamid Errachid and Nicole Jaffrezic-Renault

Supplementary material

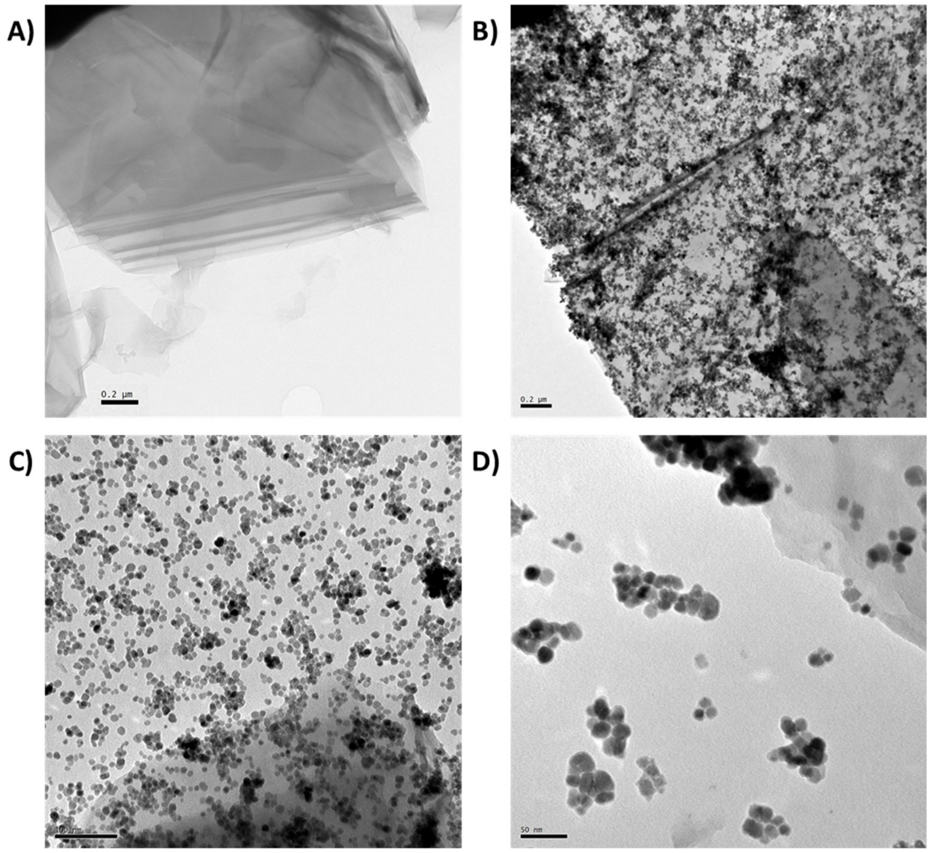

Figure S1. TEM images of A) GO (scale of 200 nm), and GO@Fe_3O_4 with a scale of B) 200 nm, C) 100 nm and D) 50 nm.

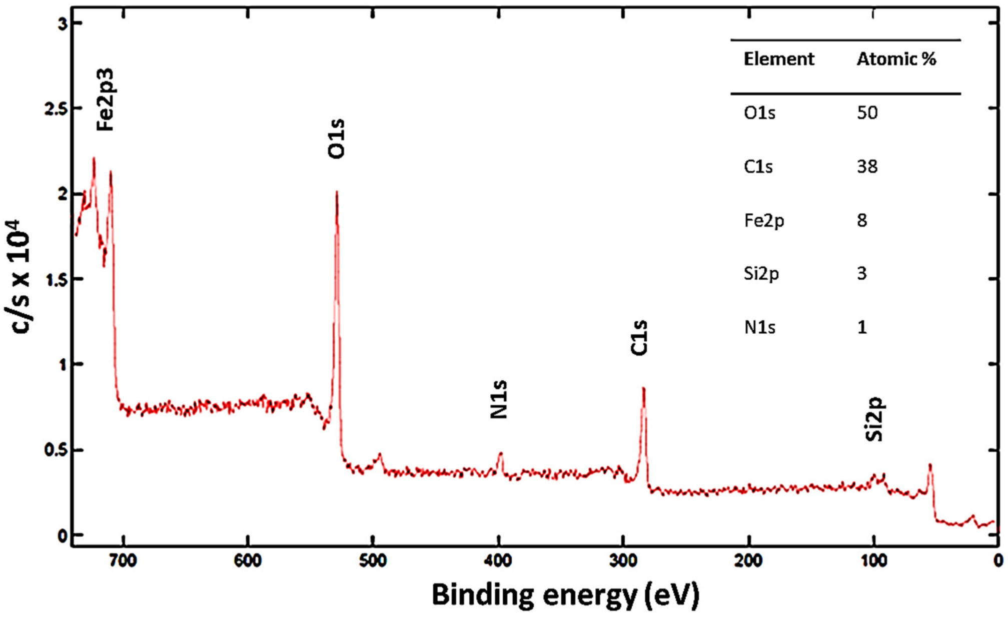

Figure S2. XPS spectra of GO@Fe_3O_4 and atomic composition.

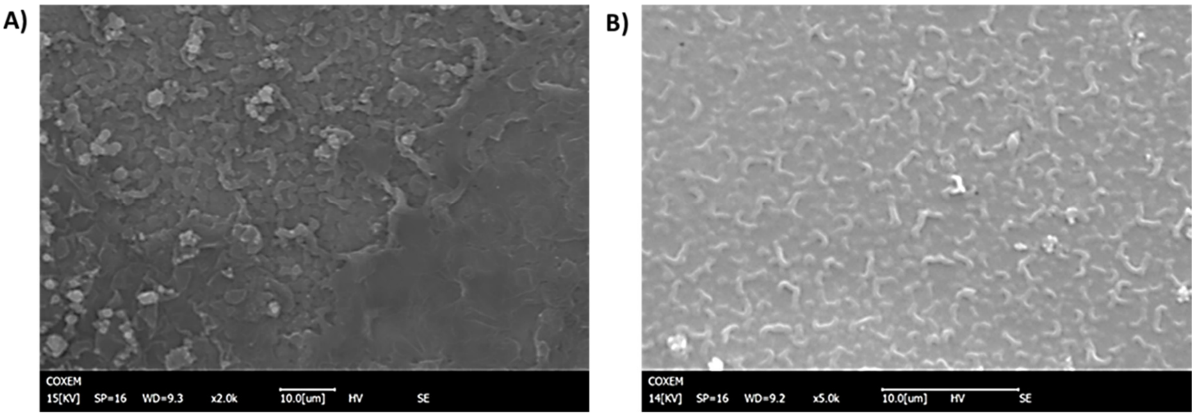

Figure S3. SEM images of PPy synthesized by electropolymerization A) prior to scan-rate optimization and b) under scan-rate optimized conditions (0.15 V/s).

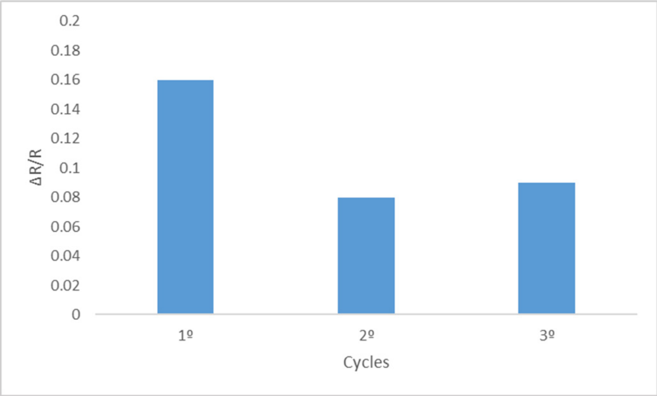

Figure S4. Reusability study results after three LYS 10 pg/mL incubation cycles.

Table S1. Average data for equivalent circuit of MIPPy/GO@Fe_3O_4 impedimetric responses

**MIP [LYS] fg/mL**	**Rs**	**CPE**	**R_{CT}**	**W**	**ΔRCT**	**±**
0	200.9	7.94E-09	228.2	63.8	-	
1.00E+01	205.7	1.19E-08	213.1	151.8	5.95	0.59
1.00E+02	199.8	1.14E-08	205.6	219.5	7.94	1.54
1.00E+03	205.5	6.10E-08	207.8	242.2	9.60	0.94
1.00E+04	199.9	5.88E-08	201.8	298.4	10.46	1.72
1.00E+05	203.8	4.63E-08	202.6	369.4	11.046	1.08
1.00E+06	205.6	2.44E-08	199.8	450.5	11.49	0.82
1.00E+07	205.8	1.55E-08	196.7	524.4	12.55	1.76
1.00E+08	204.51	1.24E-08	194.1	566	13.51	2.02
**MIP [BSA] fg/mL**	**Rs**	**CPE**	**R_{CT}**	**W**	**ΔRCT**	**±**
0	426.55	3,69E-06	2.13E+09	2.12E+13	-	
1.00E+03	442.4	3,34E-06	7.04E+06	3.10E+13	81	0.34
1.00E+05	446.4	2,97E-06	4.52E+05	1.09E+11	311	0.80
1.00E+07	433.15	3,04E-06	1.83E+05	2.39E+10	55.8	0.47
**MIP [PEROX] fg/mL**	**Rs**	**CPE**	**R_{CT}**	**W**	**ΔRCT**	**±**
0	527.35	2.3384E-06	1.89E+05	9.47E+10	-	
1.00E+03	483.7	2.9059E-06	7.28E+06	1.12E+12	5300	17.67
1.00E+05	503.65	1.6976E-06	1.92E+09	4.52E+11	483	0.35
1.00E+07	483	2.02115E-06	1.16E+06	1.24E+11	780	0.78
